# Predictors of Child’s Health in Pakistan and the Moderating Role of Birth Spacing

**DOI:** 10.3390/ijerph19031759

**Published:** 2022-02-03

**Authors:** Muhammad Farhan Asif, Salima Meherali, Ghulam Abid, Muhammad Safdar Khan, Zohra S. Lassi

**Affiliations:** 1 Department of Economics, National College of Business Administration and Economics, Lahore 54000, Pakistan; 2 Faculty of Nursing, University of Alberta, Edmonton, AB T6G, Canada; meherali@ualberta.ca; 3 Department of Business Studies, Kinnaird College for Women, Lahore 54000, Pakistan; dr.ghulamabid@gmail.com; 4 Department of Urology, King Edward Medical University, Lahore 54000, Pakistan; drsaldera@yahoo.com; 5 Robinson Research Institute, the University of Adelaide, Adelaide, SA 5005, Australia; zohra.lassi@adelaide.edu.au

**Keywords:** role of birth spacing, mother’s health care services utilization, predictors of child’s health, Pakistan

## Abstract

There is a consensus that better health should be viewed both as a means and an end to achieve development. The level of development should be judged by the health status of the population and the fair distribution of health services across the people. Many determinants affect a child’s health. This study aimed to explore a child’s health predictors and the moderating role of birth spacing on the association between mother’s health care services utilization (MHCSU) and a child’s health. In this study, we used the dataset of Pakistan Demographic and Health Survey 2017-18 to explore the predictors of child health and the moderating role of birth spacing through binary logistic regression, using SPSS version 20. The results showed an association of mother’s age (35 to 49 years), her education (at least secondary), health care services (more accessible), father’s education (at least secondary), their wealth status (high), and exposure to mass media to improved child health. However, the effect of a mother’s employment status (employed) on her child’s health is significant and negative. The coefficient of moderation term indicated that the moderating role of birth spacing on the association between MHCSU and a child’s health is positive. We conclude that birth spacing is a strong predictor for improving a child’s health. The association between MHCSU and child’s health is more distinct and positive when the birth spacing is at least 33 months.

## 1. Introduction

Children are considered the future human resource of any country, and there is a universal consensus on this [1]. Therefore, children’s health needs strong assurance for the betterment of a nation. Although there have been numerous advancements in medical technology and management, nations are still struggling to improve their rates of child morbidity and mortality [2]. Child mortality has decreased significantly in developing countries over the previous two decades, from 87 to 51 deaths per 1000 live births [3]. Children with poor health are considered to be in immense danger; therefore, it is crucial to identify the conceivable causes of health inequalities among children. Social determinants, i.e., age, residence place, parental education, exposure to mass media, etc., can play a vital role in forming desired health policies [4].

Social determinants of health have received considerable attention in the last three decades to explain the health inequalities present in a population [5]. This needs better medical care, as current outcomes are inadequate to solve the stated inequality regarding child health [6]. Consequently, other factors, such as socioeconomic status, family composition (either joint or nuclear), and race/ethnicity, need to be explored as potential predictors of a child’s health [7].

Children are more vulnerable than adults, and the poor economic status of a household impacts child health negatively through lack of awareness, malnutrition, lack of health services, and unhygienic conditions. Poor child health can lead to morbidity [8]. Although child mortality has declined in recent decades in low- and middle-income countries (LMICs), many LMICs are still struggling to reach the targets set by the Sustainable Development Goals (SDGs) [3,9]. In Pakistan, infant and under-five mortality rates are still high, i.e., 57 deaths per 1000 live births and 65.2 deaths per 1000 live births in 2021, respectively [2,10]. Thus, the gap between the current situation and the target set by the SDGs warrants investigation to determine the factors responsible for the high burden of child morbidity and mortality.

One of the important determinants of child health is socioeconomic status (SES), which is directly linked with their household’s income. The literature has stated that children who belong to less wealthy households face several health issues, for example, injuries, lung problems, etc. [11,12,13]. In contrast, children from wealthier families can afford and take high-quality foods and hygiene services that prevent them from experiencing health-related problems [14]. Income alone does not determine the health condition of children; many other aspects of SES have been examined in earlier studies [15]. Herd et al. [16] stated that education plays an important role in reducing child health issues compared to income. More educated mothers have more knowledge and awareness of child health than mothers with less education [17]. Furthermore, both parents’ education is associated with the occurrence of illnesses, infections, and deprived nutrition in children [18].

The father’s education plays a key role in the financial strength of his family and the provision of necessary health facilities [19]. It is considered that these fathers are more aware and knowledgeable, which can determine a child’s health status and if the child requires checking and treatment. The effect of women’s working status on maternal and child health service uptake has been inconsistent across studies. Some studies that exhibit a positive association state that women’s employment enhances their influence over earned money, empowering them to seek maternal and child health services [20]. Similarly, if a mother is not in good health during pregnancy and/or the fetus is exposed to teratogens, such as infectious agents, radiation, chemical agents, hormones, maternal diseases, and nutritional deficiencies, the child is more likely to experience health difficulties or death [21]. Additionally, exposure to mass media plays an important role in improving mother and child health. Television programs provide information regarding child vaccination and proper health facilities that must be provided to the children. Because of this awareness, mothers can take good care of their children [22]. 

Birth spacing and mother’s health are major predictors of child health [23]. An adequately spaced birth, i.e., between 2–4 years, improved infant survival by 2.4 times and child survival by 2.9 times [10]. Additionally, infants born small for their gestational age are twice as likely to not survive the neonatal period compared to children with an average size at birth [23]. This study examines the determinants of a child’s health and the moderating role of birth spacing on the relationship between mother’s health care services and child health in Pakistan. Cross-sectional data of from the Pakistan Demographic and Health Survey (PDHS) 2017-18 has been analyzed. The specified association has hardly been seen in the literature in the case of Pakistan.

## 2. Materials and Methods

### 2.1. Data Source

The PDHS 2017-18 dataset was obtained and utilized for analysis. The total sample size consisted of 50,450 women. Around 2264 of the 50450 women reported their child’s health variable information. We applied the listwise deletion method for removing missing data. In this method, an entire record is excluded from analysis if any single value is missing. The information on 2246 women was analyzed after removing the participants with missing data.

### 2.2. Variables and Measurement

CH = f(M.Age, M.Edu, M.Emp, MHCSU, WSH, F. Edu, EMM, BS, MHCSU * BS)

where, to examine the determining factors of child health, the functional form of the model used is given in Table 1.

The descriptive statistics provide comprehensive information on the socio-economic determinants of the children. Their frequency and percentage were calculated. Since the dependent variable was categorical, binary logistic regression was applied to examine the factors associated with child health. The dependent variable is dichotomous, i.e., the child is healthy or the child is unhealthy. The analysis of moderation allows investigating the impact of the third variable (Z) on the association among the predictor (X) and criterion variables (Y). It reveals in whom, when, and under what conditions a relation will last. Simply, it explores when and under what condition an impact occurs. We draw an arrow from the moderator to the relation between an independent and dependent variable in a moderation relationship. The moderating variable can weaken, strengthen, or reverse the nature of the existing relationship. In other words, a moderator affects the strength, direction, or even the existence of a link between study variables [27,28,29]. 

## 3. Results

The descriptive statistics (Table 2) detail the frequency of socio-economic and demographic characteristics of children. Four-fifths (81.1%) of the mothers were younger than 35, and an equal proportion (81.4%) of the total population considered their child healthy. The majority of the mothers had attained at least secondary education (71.3%), were unemployed (86.2%), rated more accessible for health care services (88.5%), had exposure to mass media (82.2%), and had births spaced for less than 33 months (72.7%). The majority of the mothers had husbands with at least secondary education (78.8%), and they belonged to the higher wealth quintile (65.0%). 

The results of the logistic regression (Table 3) demonstrate that the effect of mother’s age (β = 0.195, *p* < 0.05), her education (β = 0.115, *p* < 0.05), her access to health care services (β = 0.206, *p* < 0.05), wealth status of the household (β = 0.263, *p* < 0.05), father’s education (β = 0.197, *p* < 0.05), and exposure to mass media (β = 0.034, *p* < 0.05) on child health is positive and significant. Meanwhile, the effect of birth spacing (β = 0.179, *p* < 0.10) on child health is positive and insignificant. Birth spacing is one of multiple important factors that affect child health. In this regard, we used an interaction term of birth spacing with MHCSU. In contrast, the effect of mother’s employment status (β = −0.199, *p* < 0.05) on child health is negative and significant. 

In the present study, we are interested in understanding how changes in an MHCSU affect a child’s health. Here, we expected that birth spacing can affect this relationship, and wanted to fit a regression model in which we could use the MHCSU to predict child health. We suspect that a better utilization of mother’s health care services is associated with good child health. However, this relationship is moderated by birth spacing. An extra utilization of mothers’ healthcare services may cause better child health for women with greater birth spacing than women with fewer months of birth spacing.

The moderation analysis can be investigated by interacting (multiplying) both the variables, i.e., predictor and moderator. A common and straightforward way of exploring whether a moderating impact occurs involves adding a (regression analysis) interaction term in a multiple regression model. The result endorses the interaction effect of birth spacing on the association between MHCSU and a child’s health. We found a positive and significant interaction between MHCSU and birth spacing on child health (β = 0.236, *p* < 0.05). We plotted the interaction effects to demonstrate the moderating impact of high and low birth spacing. 

**Figure 1:**
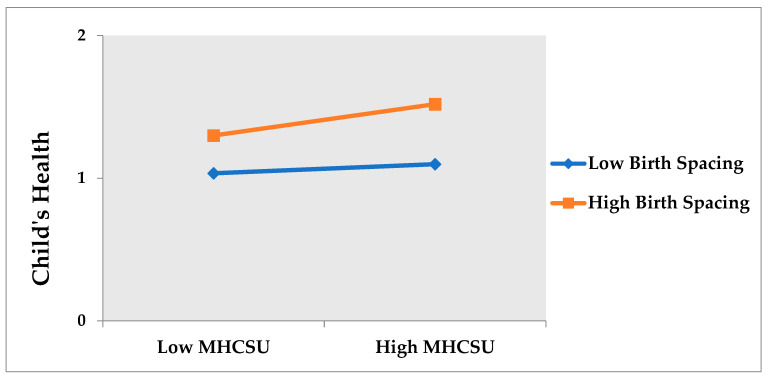
Graphical representation of moderation analysis.

The moderating effect can be easily depicted and explained by plotting the simple slopes of the interaction. We plot the relationship between predictor and criterion at low (−1 SD: standard deviation from the mean) and high (+1 SD: standard deviation from the mean) moderator values. As shown in Figure 1, the association between mother’s health care services utilization and child’s health is much more distinct and positive when the birth spacing is higher than lower birth spacing. On the other hand, child health is better when the mother takes all maternal health care services and maintains high birth spacing. 

## 4. Discussions

Using binary logistic regression, we empirically investigated the significant correlations of child health in Pakistan. Mother’s age, education, employment status, mother’s health, wealth status of their household, father’s education, exposure to mass media, and birth spacing have been identified as some of the important correlates of child health. The association between a mother’s age and a child’s health is positive. The majority of earlier studies indicate that older women utilize more maternal health services, and their children are healthier than those of younger women [28,29]. 

In contrast, another study indicates that women at a younger age are more likely to receive antenatal care, assisted delivery, and postpartum care than older women. A major reason for this is the correlation between age and the number of children (parity). Therefore, younger women are more likely to experience early birth because of social and cultural pressures attached to the country [29]. Parental education (mother’s and father’s education) is an important factor affecting child health. Educated parents are more aware of health issues and the availability of health care services and facilities, and they are therefore more likely to utilize maternal health services. Our findings confirm the results of the previous studies [29,30].

The coefficient of child’s health is negative for employed women. Employed women are said to be overburdened with domestic work, with their low paid work restricting their uptake of health services [31,32]. The effect of women’s working status on maternal and child health service uptake has been inconsistent across studies. Multiple studies that exhibit a positive association state that women’s employment enhances their influence over earned money, empowering them to seek maternal and child health services [33,34]. Studies that have established a negative association between healthcare services and employment status state that employed women face time constraints, which diminish their uptake of health services. The findings of our study support the findings of earlier research [32,35,36,37]. Another reason is that women’s working status in developing countries is usually poverty-induced [38]. Similarly, women employed in unorganized sectors are low-paid, which does not enhance their economic betterment [39]. Furthermore, the uptake of health services depends on the nature of the working status of women.

Healthy mothers have healthy children. Poor health of the mother (malnutrition) can contribute to developmental complications across childhood [40]. If a mother is not in good health during the pregnancy and/or the fetus is exposed to teratogen(s) such as infectious agents, radiation, chemical agents, hormones, maternal diseases, and nutritional deficiencies, the child is more likely to experience health difficulties or death [22]. Ensuring that the expecting mother has access to an acceptable diet will ensure that the children have adequate nutrients to grow and develop as a healthy fetus [41].

Higher income is considered the strongest factor affecting child health [42]. Child health is observed to be better in affluent households as mothers have more access to health services, and hence can have greater ability to utilize maternal and child health services [43]. Women’s exposure to media can also be an important indicator of maternal and child health service uptake [44,45]. Exposure to media (such as newspapers, radio, TV) provides women with information about healthcare issues, family planning, etc., enabling them to use such services when they need them [46,47,48]. Birth interval span is linked with child health and child mortality [49,50,51,52], and deprived antenatal results have long-term effects on socio-economic achievement [53,54] and health [55,56,57,58,59], even in developed economies.

Moreover, short birth spacing can increase sibling rivalry and dilute the means and time that parents can invest in their children [60]. On the other hand, adequately spaced birth has a certain importance for a child’s survival. A previous study explains that when the length of birth spacing is less than two years, the newborn baby, on average, is two times as likely to die in infancy than a child born after a longer birth spacing [61].

The World Health Organization has recommended a minimum interval of 33 months between births, or at least 24 months before attempting a subsequent pregnancy, in order to reduce the risk of adverse maternal, perinatal, and infant outcomes. Short pregnancy interval does not enable the mother to fulfill the nutritional requirements, particularly folate and iron, for the subsequent pregnancy. These nutritional depletions are not only harmful to maternal health but also to child health. Similarly, short pregnancy intervals are associated with small gestational age birth, preterm birth, low birth weight, stillbirth, child mortality, and maternal mortality [26].

The results are extracted from the data provided by the mother respondents belonging to the age group of 15–49. The mother’s empowerment regarding their child’s health care, alongside socioeconomic characteristics, greatly influenced their child’s health and child health-related issues. Further empirical support will be helpful to establish the significance of the prevailing factors of the child’s health. 

## 5. Recommendations

In light of the results from this paper, a significant and steady increase in investment in health and education is crucial to reduce maternal and child mortality and achieve the SDGs by 2030. Education and employment boost awareness and confidence, improving the livelihood and health of both the family and society at large. Mass media and the digital space play a significant role in this shift; therefore, governments should provide means to support girls’ education and launch different programs to support the efforts of households in controlling the size of their families so that they can ensure a higher quality of living standard for their children and play the active role of parents to their best ability.

Governments should also focus on improving maternal health care utilization and providing means to increase the uptake of antenatal, natal, and postnatal care that ensures the health of the mother and the child. The provision of maternal and child healthcare through outreach services and mass media to increase awareness about the importance of birth spacing is crucial in the country. There should be routine research on maternal and child health and family planning to identify the issues earlier and implement the necessary remedial programs.

## 6. Conclusions

The results conclude that mother’s age, education, and employment status, mother’s health care services utilization, household wealth status, father’s education, exposure to mass media, and birth spacing have been identified as important factors of child health. The odds ratio indicates that children of older women aged 35–49 were healthier than those of younger women aged 15–34 years. The odds ratio shows that children of higher-educated mothers were healthier than children of less-educated mothers. Children whose mothers were accessing proper health care services were more likely to be healthier than those whose mothers accessed less health care services. The results indicate that the likelihood of a child’s health increases with an increase in the wealth status of their mother’s household, their father’s education, exposure to mass media, and adequate birth spacing. At the same time, the odds ratio indicates that the children of employed mothers were unhealthy compared to those of unemployed mothers. The outcome of the interaction term suggested that the association between MHCSU and a child’s health is more distinct and positive when the birth spacing is at least 33 months. 

## Figures and Tables

**Table 1:** Variables and Measurement.

Symbol	Variables	Definition of Variables	Measurement
CH	Child’s Health	Child weight at birth has been used as a proxy of child health. If a child’s weight at birth is less than 2.5 kg, this is considered unhealthy, and if a child’s weight at birth is at least 2.5 kg, this is considered healthy.	1 = Unhealthy child 2 = Healthy child
M.Age	Mother’s Age	Mother’s age has been classified into two different groups: mothers aged 15–34, and mothers aged 35–49 years.	1 = 15–34 2 = 35–49
M.Edu	Mother’s Education	Mother’s education is divided into two categories. If the mother has no education and has attended primary school only, this is considered as “less than secondary,” and if the mother has completed secondary school education and has completed higher education, then this is considered as “at least secondary.”	1 = Less than secondary 2= At least secondary
M.Emp	Mother’s Employment Status	Mother’s employment status is divided into two categories, i.e., mothers currently not working, and mothers currently working.	0 = Currently not working (unemployed) 1 = Currently working (employed)
MHCSU	Mother’s Health Care Services Utilization	This variable was constructed based on whether the mother had at least 4 or more antenatal care visits (ANC), skilled birth attendance (SBA), and received postpartum care within 42 days of delivery. If the mother had received all these services, this is considered more accessible, and if the mother had not received all these services, this is considered less accessible. We used ANC, SBA, and postpartum care as indicators to measure maternal health care utilization, which had been used in several earlier primary studies from neighboring countries [24,25].	1 = Less accessible 2 = More accessible
WSH	Wealth Status of Household	Wealth status was divided into quintiles from poorest to richest. Women belonging to the poorest, poorer, and middle quintiles are considered low wealth status, and if women belonging to the richer and richest quintiles are considered high wealth status.	1 = Low wealth status 2 = High wealth status
F.Edu	Father’s Education	Father’s education is divided into two categories. If the father has no education and has attended primary school, this is considered “less than secondary,” and if the father has completed secondary school or higher education, this is considered “at least secondary.”	1 = Less than secondary 2= At least secondary
EMM	Exposure to Mass Media	The PDHS 2017-18 provides information on households’ ownership of a radio or television, along with the type of health message delivered to women through these media. In this study, the presence of a television (TV) in the household has been used as a proxy for this variable.	1 = Presence of TV 0 = Otherwise
BS	Birth Spacing	The World Health Organization has recommended a minimum birth interval of 33 months between two births, or at least 24 months before attempting the subsequent pregnancy, to reduce the risk of adverse maternal, perinatal, and infant outcomes [26].	1 = Women take less than 33 months birth spacing between two children 2 = Women take at least 33 months birth spacing between two children
MHCSU * BS	Mother’s Health Care Services Utilization * Birth Spacing	The interaction term of mother’s health care services utilization and birth spacing. The interaction term has been used to examine the moderating effect of birth spacing on the relationship between a mother’s health care services utilization and a child’s health.	

**Table 2:** Socio-economic and demographic determinants of children.

Socio-Economic Characteristics		Frequency	Percentage (%)
Child’s Health	Unhealthy	415	18.5
	Healthy	1831	81.5
Mother’s Age	<35 years	1821	81.1
	>35 years	425	18.9
Mother’s Education	Less than secondary	643	28.6
	At least secondary	1603	71.4
Mother’s Employment Status	Unemployed	1938	86.3
	Employed	308	13.7
Mother’s Health Care Services Utilization	Less accessible	257	11.4
	More accessible	1989	88.6
Wealth Status of Household	Low	787	35.0
	High	1459	65.0
Father’s Education	Less than secondary	464	20.7
	At least secondary	1782	79.3
Exposure to Mass Media	No	397	17.7
	Yes	1849	82.3
Birth Spacing	Less than 33 months	1633	72.7
	At least 33 months	613	27.3

**Table 3:** Results of Binary Logistics Regression.

Independent Variables		Β	*p*-Value	Odds Ratio	95% C.I. for EXP(β)	
					Lower	Upper
Constant		0.749	0.000	2.115		
Mother’s Age	<35 years	Reference				
	>35 years	0.195 *	0.013	1.215	0.863	1.592
Mother’s Education	Less than secondary	Reference				
	At least secondary	0.115 *	0.039	1.122	0.835	1.298
Mother’s Employment Status	Unemployed	Reference				
	Employed	−0.199 *	0.020	0.819	0.587	1.171
Mother’s Health Care Services Utilization	Less accessible	Reference				
	More accessible	0.206 *	0.011	1.500	1.091	1.916
Wealth Status of Household	Low	Reference				
	High	0.263 *	0.045	1.301	1.116	1.672
Father’s Education	Less than secondary	Reference				
	At least secondary	0.197 *	0.016	1.218	0.797	1.583
Exposure of Mass Media	No	Reference				
	Yes	0.034 *	0.021	1.014	0.851	1.396
Birth Spacing	Less than 33 months	Reference				
	At least 33 months	0.179	0.058	1.196	0.827	1.528
Mother’s Health Care Services Utilization * Birth Spacing		0.236 *	0.034	1.279	0.831	1.729

* *p* < 0.05.

## Data Availability

We have used the secondary data of PDHS 2017–2018. Available at: https://www.nips.org.pk/study_detail.php?detail=MTgw (accessed on 13 May 2021).

## List of Abbreviations

ANC	Antenatal care
LMICs	Low- middle-income countries
MHCSU	Mother’s health care services utilization
PDHS	Pakistan Demographic and Health Survey
SBA	Skilled birth attendance
SDGs	Sustainable Development Goals
SES	Socio-economic status
TV	Television
UMNFP	Unmet Need for Family Planning
USA	United States of America
